# Field Evaluation of Picaridin Repellents Reveals Differences in Repellent Sensitivity between Southeast Asian Vectors of Malaria and Arboviruses

**DOI:** 10.1371/journal.pntd.0003326

**Published:** 2014-12-18

**Authors:** Karel Van Roey, Mao Sokny, Leen Denis, Nick Van den Broeck, Somony Heng, Sovannaroth Siv, Vincent Sluydts, Tho Sochantha, Marc Coosemans, Lies Durnez

**Affiliations:** 1 Department of Biomedical Sciences, Institute of Tropical Medicine, Antwerp, Belgium; 2 National Center for Parasitology, Entomology and Malaria Control, Phnom Penh, Cambodia; 3 Malaria Molecular Epidemiology Unit, Institut Pasteur du Cambodge, Phnom Penh, Cambodia; The Rockefeller University, United States of America

## Abstract

Scaling up of insecticide treated nets has contributed to a substantial malaria decline. However, some malaria vectors, and most arbovirus vectors, bite outdoors and in the early evening. Therefore, topically applied insect repellents may provide crucial additional protection against mosquito-borne pathogens. Among topical repellents, DEET is the most commonly used, followed by others such as picaridin. The protective efficacy of two formulated picaridin repellents against mosquito bites, including arbovirus and malaria vectors, was evaluated in a field study in Cambodia. Over a period of two years, human landing collections were performed on repellent treated persons, with rotation to account for the effect of collection place, time and individual collector. Based on a total of 4996 mosquitoes collected on negative control persons, the overall five hour protection rate was 97.4% [95%CI: 97.1–97.8%], not decreasing over time. Picaridin 20% performed equally well as DEET 20% and better than picaridin 10%. Repellents performed better against *Mansonia* and *Culex* spp. as compared to aedines and anophelines. A lower performance was observed against *Aedes albopictus* as compared to *Aedes aegypti*, and against *Anopheles barbirostris* as compared to several vector species. Parity rates were higher in vectors collected on repellent treated person as compared to control persons. As such, field evaluation shows that repellents can provide additional personal protection against early and outdoor biting malaria and arbovirus vectors, with excellent protection up to five hours after application. The heterogeneity in repellent sensitivity between mosquito genera and vector species could however impact the efficacy of repellents in public health programs. Considering its excellent performance and potential to protect against early and outdoor biting vectors, as well as its higher acceptability as compared to DEET, picaridin is an appropriate product to evaluate the epidemiological impact of large scale use of topical repellents on arthropod borne diseases.

## Author Summary

Malaria and arboviruses are transmitted by several mosquitoes. Targeting these mosquitoes instead of the pathogens can contribute to prevention of these diseases. For mosquitoes biting throughout the night, mosquito nets (preferably impregnated with insecticides) are very effective for mosquito control. However, bites of day-, evening- and outdoor-biting mosquitoes have to be prevented in different ways, for example by applying repellents on the skin which contain DEET or other active ingredients such as picaridin. Here we report on the evaluation of the performance of two formulated picaridin repellents (lotion 10% and spray 20%) against mosquito bites, including vectors of arboviruses and malaria in the field in Cambodia. These repellent formulations were compared to a DEET solution (20%). In general, all repellents performed very well, providing more than 97% protection against mosquito bites when used for five consecutive hours. At the highest concentration, the picaridin repellent performed similarly to DEET. However, different mosquito species reacted differently to the repellents. As such, repellents can provide an additional protection against bites of malaria and arbovirus vectors.

## Introduction

Vector-borne diseases remain major contributors to the burden of diseases in the tropics [1], [2]. The most important vectors for transmission of diseases are bloodsucking arthropods, and especially mosquitoes. Worldwide, about 3500 mosquito species have been described, but only a few of them are able to transmit human disease. The mosquito-borne diseases of public health importance include malaria, filariasis, and arboviral diseases such as dengue, chikungunya, Japanese encephalitis, and yellow fever [1], [3]. For these diseases, targeting the mosquito instead of the pathogen contributes greatly to disease prevention. Current vector control programs are primarily based on insecticides [1], [4]. For malaria, which is one of the most serious vector-borne diseases affecting millions of people, upscaling of vector control programs has greatly contributed to its worldwide decrease, and especially in Southeast Asia substantial progresses have been observed [5]. The present vector control programs are primarily based on the distribution of long-lasting insecticidal nets (LLINs) and/or application of indoor residual spraying (IRS). However IRS has little impact on outdoor resting vectors, and outdoor and/or early biting species are not affected by LLINs [4]. Some vector species, such as *Anopheles arabiensis* in Africa [6], *Anopheles maculatus* and *Anopheles dirus* in Asia [7], [8], or *Aedes aegypti* and *Aedes albopictus* are then less or not vulnerable to one of these two preventive methods. As such, in Southeast Asia, residual malaria transmission due to outdoor and early biting malaria vectors constitutes an important, but often neglected, public health concern in some provinces of each country [9]. Vector control is also of high importance in preventing arboviruses such as dengue (*Flaviviridae*) and chikungunya (*Togaviridae*) as no treatment or vaccine is available [10]–[12]. However, both viruses are transmitted by the day-and outdoor-biting mosquitoes *Ae. aegypti* and *Ae. albopictus* [3], [13]. As early and outdoor biting proportions of vectors will maintain malaria and arbovirus transmission, there is an urgent need for additional control measures tackling these fractions of the vector population [4]. Synthetic repellents are a common means of personal protection against mosquito bites. N, N-diethyl-3-methylbenzamide (DEET) is the most commonly used active ingredient in commercially available repellents and has gained wide acceptance in the western world [14]. Another promising synthetic repellent, which was developed by Bayer in the 1980s using molecular modelling, is 1-piperidinecarboxylic acid, 2-(2-hydroxyethyl)-,1-methylpropylester (commonly known as picaridin). In contrast to DEET, picaridin does not dissolve plastics and other synthetics (coatings, sealants), and is biodegradable. Moreover it is cosmetically more acceptable (skin feeling, odour) than DEET [in 14]. The effectiveness of this repellents has shown to equal DEET [15]–[20], or be better than DEET [21].

Different studies demonstrate the efficacy of topical repellents as personal protection tool against malaria [22]–[26], whereas others fail to prove such an effect [27]. There is currently no evidence available for repellents as a community protection tool that decreases transmission. The epidemiological efficacy and the impact of topical repellents on malaria and arbovirus transmission will depend on two major factors, which are the performance of the repellent to protect an individual from getting bitten by a mosquito, and the adherence/coverage to repellent treatment in the study community [28]. As such, for implementing the use of repellents in malaria and arbovirus control programs, knowledge on the entomological efficacy of specific repellents is a prerequisite. In Cambodia, a large scale study to raise evidence on the effectiveness of mass use of effective and safe repellents (picaridin) in addition to insecticide impregnated bed nets in controlling malaria and arbovirus infections was conducted (MalaResT project, trial registered as NCT01663831). Present study explores in field conditions the protective efficacy of two formulations of picaridin against the bites of Southeast Asian mosquitoes. A protocol adapted from the World Health Organisation Pesticides Evaluation Scheme guidelines for efficacy testing of mosquito repellents for human skin [29] was applied in which human landing collections were carried out on volunteers applying either a placebo or a test repellent on their exposed limbs. The efficacy and performance of the two formulations of picaridin (lotion 10% and spray 20%) were assessed and compared to an ethanol solution of 20% DEET over a period of two years.

## Materials and Methods

### Study area & surveys

The study was carried out in two malaria endemic provinces in Cambodia, namely Mondolkiri (two villages: Krang Tes (latitude 12.636354N, longitude 107.348258E) and Pou Siam (latitude 12.340183N, longitude 107.148045E)) and Pailin (1 village: Kngok (latitude 12.919693N, longitude 102.676803E)), that were chosen based on previous knowledge on the presence of *An. dirus* s.l. or *Anopheles minimus* s.l.. As no *An. minimus* s.l. were collected in Pou Siam, collections were stopped in this village after two surveys, and this village was replaced by Kngok (Pailin).

A total of 8 surveys were organized during which mosquito collections took place during 10 days. Pou Siam was only included in surveys 1 and 2, and Kngok in surveys 3 to 7, whereas Krang Tes was included in all surveys. In Krang Tes, the study setup was duplicated as from survey 3 onwards. The surveys took place in May, July, September, and November of 2012 and 2013. In each of the villages, 5 outdoor collection points, near to houses, were chosen which were at least 20 meters apart to avoid mosquito diversion between treated and negative control persons [30]. The protocol of the study was adapted from the WHOPES guidelines for efficacy testing of mosquito repellents on human skin [29].

### Repellent treatments

Five treatments were included in the study: two negative controls (ethanol), one technical grade DEET treatment used as a positive control, given that this repellent is considered as the golden standard (from Acros Organics diluted at 20% in ethanol), and two formulations of picaridin (10% repellent lotion and 20% repellent spray formulated by S.C. Johnson). The picaridin formulated products complied with the WHO specifications (confirmed by the chemical analysis at CRA-W, Gembloux, certificate of analysis ITM/FO 23005/Ch.5362 to 5365/2012/A). An experimental replicate consisted of 5 consecutive days during which the lower limbs of 5 persons were treated with repellents or ethanol, followed by mosquito collections on the treated limbs during 5 consecutive hours. This experimental replicate was repeated 46 times over the 8 surveys. The effects of day of treatment, collection site and test person were accounted for by following a 5×5×5 Graeco-Latin Square rotation design. Each day, one of the 5 test persons was assigned to one of the treatments, and the collection sites were rotated among the test persons each hour.

Before application of the treatments, the legs of the test person were washed with unscented soap, followed by rinses with clean water and ethanol. The treatments were applied on both legs, between ankle and knee at 1 ml/600 cm^2^. Test persons wore long-sleeved shirt, long trousers, and socks up to the ankle. The legs of the trousers were rolled up to the knee to expose only the treated part of the legs to biting mosquitoes. After finishing the test session, the limbs were washed again.

### Mosquito collections, identification and laboratory analysis

Human landing collections were performed starting 30 minutes after treating the legs of 5 trained volunteers, between 17 h and 22 h, except for the last survey, during which collections took place between 19 and 24 h (but also 30 minutes after treatment of legs). There was a continuous exposure to mosquitoes, with a break of 15 minutes at the end of each hour so as to allow the test persons to rest and change collection site. Specimens were collected in labelled individual glass tubes and identified in the field at species level based on morphological characters using identification keys as described in [8]. For *An. dirus* s.l., *An. minimus* s.l., *An. maculatus* s.l., *Anopheles barbirostris* s.l., *Ae. aegypti*, and *Ae. albopictus*, the parity was determined by examination of the tracheoles within the ovaries in the field [31]. For long-term storage, all mosquitoes were kept dry, in an individual plastic capsule by specimen with the corresponding label.

Head and thorax of all anophelines were analysed by the ELISA method for detection of the circumsporozoite protein (CSP) as described in [32]. All ELISA positive specimens were subjected to a *Plasmodium* specific PCR [32], as false positivity was previously observed in this region. Molecular species identification was performed for mosquitoes morphologically identified as *An. dirus* s.l., *An. minimus* s.l. and *An. maculatus* s.l. as described previously [8].

### Data entry & analysis

All data were collected on standard forms, and were double-entered in a pre-tested Access database by two independent data entry clerks. Databases were compared by using Epi Info ™ 3.5.3, and inconsistencies were checked with the hard copy forms and corrected.

Repellent efficacy was calculated as percent repellency (%R) according to the formula %R  =  ((C-T)/C)*100, Where C is the average of the total number of mosquitoes biting on the lower legs of the two individuals with the control treatment, and T is the total number of mosquitoes biting on the lower legs of a repellent-treated subject [29]. Confidence limits of proportions were calculated according to the Wilson procedure without correction for continuity as described in [33].

Generalized Linear Mixed Models using poisson or negative binomial distributions [34] and their zero-inflated variants (glmmADMB function in the glmmADMB package applied in R version 3.1.0) were fitted to the data with the daily mosquito count on the treated persons for the different treatments as dependent variable, the treatment and the mosquito genus or vector species and their interaction as explanatory variables, and survey, village, collection day, location, and collector as random factors. Mosquito counts on the treated persons were corrected for the total amount of mosquitoes collected per genus or species on the negative control persons by using the logarithm of the latter as offset in the model. Model comparison was performed by likelihood ratio tests. The final model used a negative binomial distribution, including the treatment and genus/species as fixed effects (without their interaction), and the survey, village and location (nested within village) as random effects. Incidence Rate Ratios (IRR) were calculated by exponentiation of the model coefficients and their 95% confidence interval.

For estimation of the Median Complete Protection Time of each repellent, Kaplan-Meier survival analysis was carried out for each mosquito genus and selected vector species according to [29]. For this analysis, based on the complete protection times (i.e. time until which one bite was obtained) recorded per treatment each day, only days during which individuals of the respective genus or species were collected on the negative control persons were included. For studying whether the percent repellency decreased over the five hours of collection, a Chi square for linear trend analysis was performed on the hourly aggregated data per genus or species for each repellent, by using the StatCalc function Chi Square for Trend in Epi Info 7. The Bonferroni correction was used to correct for multiple comparisons.

A logistic regression model was carried out (glm function in the stats package applied in R version 3.1.0) to study differences in parity rate between treatments and vector species. The model included the parity status of an individual mosquito as outcome (0 for nulliparous and 1 for parous), and treatment, vector species and their interaction as explanatory variables. Odds Ratios were calculated by exponentiation of the model coefficients and their 95% confidence interval.

### Ethical approval

The study protocol was approved by the ethical committees of the National Centre of Malariology CNM in Phnom Penh (Cambodia) and of the University of Antwerp/the Institute of Tropical Medicine of Antwerp (Belgium) under Belgian registration number B300201112714. The mosquito collectors were informed about the objectives, process and procedures of the study and written informed consent was obtained from them. Collector candidates were invited among the adult village population and if individuals wanted to withdraw they were allowed to do so at any time without prejudice. A Rapid Diagnostic Test for malaria diagnosis was done before the start and approximately 14 days after the end of each survey. When required, medical care was provided throughout the study.

## Results

### Mosquito biting rates

In 460 man collection evenings, a total of 5048 mosquitoes were collected on negative control persons, of which 2133 were *Culex* spp., 1169 were *Mansonia* spp., 664 were *Aedes* spp., and 1082 were *Anopheles* spp. Only *Aedes* spp. and *Anopheles* spp. were morphologically identified to species level (Table 1). Given the low number of mosquitoes collected in Pou Siam, this village was excluded from further analysis.

**Table 1:** Number of mosquitoes collected on negative control persons in each village and on treated persons per mosquito species based on morphological identification.

	Negative controls				picaridin 10%	picaridin 20%	DEET 20%
	Krang Tes (320 collection evenings)	Pou Siam (40 collection evenings)	Kngok(100 collection evenings)	Total N° on negative controls			
*Aedes* spp.*	88	10	4	102	7	5	5
*Aedes aegypti*	0	0	341	341	0	1	2
*Aedes albopictus*	151	1	69	221	14	2	3
*Anopheles* spp.*	16	0	0	16	0	0	0
*Anopheles aconitus*	15	0	3	18	0	0	0
*Anopheles annularis*	1	0	0	1	0	0	0
*Anopheles barbirostris* s.l.	10	0	85	95	15	10	1
*Anopheles culicifacies*	0	0	3	3	0	0	0
*Anopheles dirus* s.l.	61	0	0	61	3	3	1
*Anopheles hyrcanus*	85	0	0	85	2	0	2
*Anopheles indefinitus*	76	0	0	76	0	0	0
*Anopheles jamesi*	13	0	0	13	0	0	0
*Anopheles jeyporiensis*	34	0	3	37	0	0	0
*Anopheles kochi*	1	0	0	1	0	0	0
*Anopheles maculatus* s.l.	111	3	105	219	7	4	1
*Anopheles minimus* s.l.	22	0	225	247	6	1	2
*Anopheles nivipes*	6	0	0	6	0	0	0
*Anopheles philippinensis* s.l.	18	0	0	18	1	0	0
*Anopheles pseudojamesi*	29	0	0	29	2	1	0
*Anopheles splendidus*	25	0	0	25	1	0	0
*Anopheles subpictus*	112	0	0	112	2	0	0
*Anopheles tesselatus*	0	0	12	12	2	0	0
*Anopheles vagus*	2	0	0	2	0	0	0
*Anopheles varuna*	5	0	1	6	0	0	0
*Culex* spp.*	1346	2	785	2133	40	7	11
*Mansonia* spp.*	1125	36	8	1169	18	10	8

The number of collection evenings is indicated for each village.

* not identified to species level

**Figure 1:**
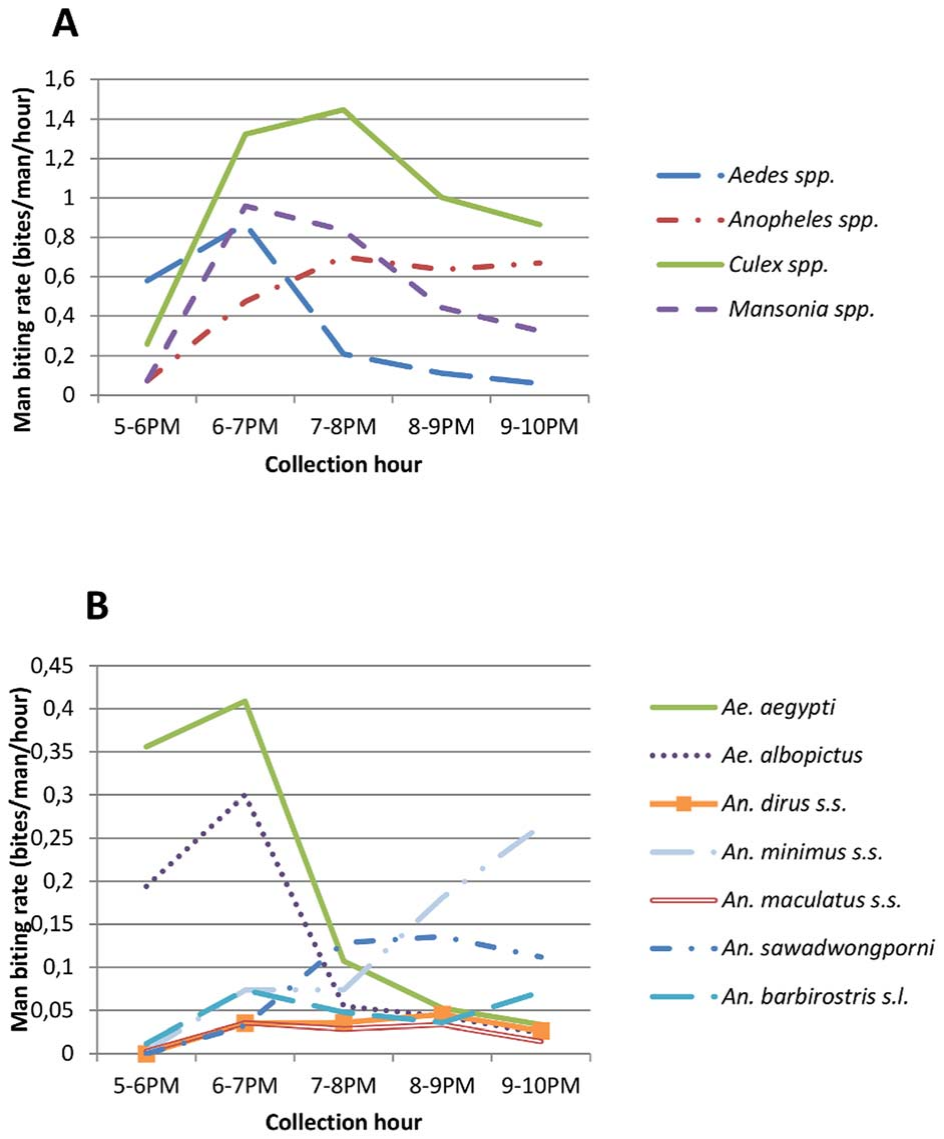
Hourly biting rate calculated as the number of bites per man per hour on negative control persons for the mosquito genera (A) and selected vector species (B).

For mosquitoes collected between 5 and 10 pm, biting peaks differed between mosquito genera, being 6–7 PM for *Aedes* spp., *Culex* spp., and *Mansonia* spp., and a steady, slightly rising man biting rate for *Anopheles* spp. from 6 to 10PM (Fig. 1A).

Main vector species *Ae. albopictus* (n = 221), *Ae. aegypti* (n = 341), *An. dirus s.s*. (n = 61, molecularly confirmed), *An. minimus s.s.* (n = 247, molecularly confirmed), *An. maculatus s.l.* (molecularly confirmed to contain *An. maculatus s.s.* (n = 48) and *An. sawadwongporni* (n = 169)), and *Anopheles barbirostris s.l.* (n = 95) were caught in sufficient numbers for the following analyses.

Between 5 and 10 PM, biting peaks differed between vectors species, being 6–7 PM for both *Ae. albopictus* and *Ae. aegypti*, 7–10 PM for *An. sawadwongporni*, 9–10 PM for *An. minimus s.s.* and *An. barbirostris s.l.*, and a slightly increasing biting rate between 6 and 9PM for *An. dirus s.s.* and *An. maculatus s.s* (Fig. 1B).

### Repellent performance

Median complete protection times were calculated to be over five hours for all mosquito genera and all vector species using Kaplan-Meier survival analysis, and could thus not be estimated as the experiment only measured repellent effectiveness for up to five hours. No significant decrease in protective efficacy was observed for the mosquito genera or vector species within the five hours of collection (S1 and S2 Figs.).

Repellent performance measured over five hours was generally high, with for all mosquito genera more than 90% of the mosquito bites prevented (S3a Fig., Table 2). Picaridin 20% (%R = 98.36% [95%CI: 97.78–98.79]) and DEET 20% (%R = 98.60 [95%CI: 98.06–98.99]) performed equally well (IRR 0.801, p = 0.517), but more mosquitoes were repelled by DEET & picaridin 20% as compared to picaridin 10% (%R = 95.36% [95%CI: 94.46–96.12]) (p<0.01 for both). This was the case for all genera, as including the interaction between treatment and genus did not improve the negative binomial model. Independent of the treatment, mosquito repellents were more effective against *Mansonia* spp. (%R = 98.00 [95%CI: 97.22–98.57]) and *Culex* spp. (%R = 98.19 [95%CI: 97.67–98.60]) as compared to *Anopheles* spp. (%R = 95.92 [95%CI: 94.84–96.78] and *Aedes* spp. (%R = 96.53% [95%CI: 95.19–97.51]) (Tables 1 and 3; S3a Fig.).

**Table 2:** Percent repellency with 95% confidence interval between square brackets for repellents, mosquito genera and mosquito species separately and for all mosquitoes and all repellents combined.

	Picaridin 10%	Picaridin 20%	DEET 20%	All repellents
**All mosquitoes**	95.36 [94.46–96.12]	98.36 [97.78–98.79]	98.60 [98.06–98.99]	97.44 [97.06–97.77]
***Anopheles*** ** spp.**	92.59 [90.07–94.51]	96.48 [94.57–97.73]	98.70 [97.34–99.37]	95.92 [94.84–96.78]
***Aedes*** ** spp.**	94.49 [91.47–96.49]	97.86 [95.65–98.96]	97.24 [94.86–98.55]	96.53 [95.19–97.51]
***Culex*** ** spp.**	96.25 [94.93–97.23]	99.34 [98.65–99.68]	98.97 [98.16–99.42]	98.19 [97.67–98.60]
***Mansonia*** ** spp.**	96.82 [95.18–98.04]	98.59 [97.13–99.34]	98.59 [97.24–99.28]	98.00 [97.22–98.57]
**All selected vectors**	92.37 [89.94–94.25]	96.44 [94.62–97.66]	98.31 [96.91–99.08]	95.71 [94.66–96.58]
***Ae. aegypti***	100.00 [97.79–100.00]	99.41 [96.74–99.90]	98.83 [95.81–99.68]	99.41 [98.29–99.80]
***Ae. albopictus***	87.27 [79.76–92.26]	98.18 [93.61–99.50]	97.27 [92.28–99.07]	94.24 [91.18–96.28]
***An. barbirostris*** ** s.l.**	68.42 [53.84–79.61]	78.95 [65.09–88.01]	97.89 [88.88–99.62]	81.75 [74.69–87.28]
***An. dirus*** ** s.s.**	90.16 [74.38–96.54]	90.16 [74.38–96.54]	96.72 [83.33–99.41]	92.35 [85.12–96.26]
***An. maculatus*** ** s.s.**	100.00 [86.20–100.00]	100.00 [86.20–100.00]	100.00 [86.20–100.00]	100.00 [94.87–100.00]
***An. sawadwongporni***	91.72 [83.79–95.91]	95.27 [88.39–98.13]	98.82 [93.56–99.79]	95.27 [91.93–97.28]
***An. minimus*** ** s.s.**	95.14 [89.76–97.70]	99.19 [95.54–99.86]	98.38 [94.26–99.55]	97.57 [95.45–98.72]

**Table 3:** Negative binomial mixed effects analysis of the effect of repellent treatment and mosquito genus on the number of mosquitoes collected per man per day.

Group 1	Group 2	IRR^a^ [95%CI]	p-value
**Treatment**			
picaridin 20%	< picaridin 10%	0.429 [0.237–0.777]	0.005
DEET 20%	< picaridin 10%	0.344 [0.184–0.642]	<0.001
DEET 20%	picaridin 20%	0.801 [0.410–1.566]	0.517
**Genus**			
*Anopheles* spp.	*Aedes* spp.	1.199 [0.639–2.252]	0.572
*Anopheles* spp.	> *Culex* spp.	2.765 [1.541–4.960]	<0.001
*Anopheles* spp.	> *Mansonia* spp.	2.511 [1.316–4.794]	0.005
*Aedes* spp.	> *Culex* spp.	2.306 [1.223–4.343]	0.010
*Aedes* spp.	> *Mansonia* spp.	2.094 [1.043–4.202]	0.038
*Culex* spp.	*Mansonia* spp.	0.908 [0.477–1.730]	0.770

Incidence Rate Ratios with 95% confidence interval and p-values are reported.

a The Incidence Rate Ratio (IRR) indicates how much more (if >1) or less (if <1) mosquitoes were collected in Group 1 as compared to Group 2. In the group with the highest number of mosquitoes collected, the protective efficacy of the tested repellents is the lowest.

Also for the vector species, the repellents performed very well, with at least 90% of the mosquitoes repelled by the repellents with higher concentration of active ingredients (DEET 20% and picaridin 20%), except for *An. barbirostris* of which only 78.95% [95%CI: 65.09–88.01%) were repelled by picaridin 20% (S3b Fig.). When modelling the protective efficacy of the repellents only for the selected vector species, similar results were observed for the comparison between repellents as for all mosquito genera: DEET 20% and picaridin 20% exhibited a higher protective efficacy (%R = 98.31% [95%CI: 96.91–99.08%] and 96.44% [95% CI: 94.62–97.66%] respectively) as compared to picaridin 10% (%R = 92.37% [95%CI: 89.94–94.25%]), and the interaction between treatment and species did not improve the model. Vector species reacted differently to the repellent treated persons (Tables 1, 2, 4), with *Ae. aegypti* (%R = 99.41% [95%CI: 98.29–99.8%]) and *An. minimus* s.s. (%R = 97.57% [95%CI: 95.45–98.72%]) being more repelled as compared to *An. dirus* s.s. (%R = 92.35% [95%CI: 85.12–96.26%]), *Ae. albopictus* (%R = 94.24% [95%CI: 91.18–96.28%]), and *An. barbirostris* s.l. (%R = 81.75% [95%CI: 74.69–87.28]). As *An. maculatus* s.s. (%R = 100% [95%CI: 94.87–100%]) was only collected on the negative control persons, and the model did not converge due to this event, this species was deleted from the analysis.

**Table 4:** Negative binomial mixed effects analysis of the effect of repellent treatment and vector species on the number of mosquitoes collected per man per day.

**Group 1**	**Group 2**	**IRR^a^ [95%CI]**		**p-value**	
**Treatment**					
picaridin 20%	picaridin 10%		0.504 [0.250–1.014]		0.055
DEET 20%	< picaridin 10%		0.246 [0.107–0.567]		<0.001
DEET 20%	picaridin 20%		0.489 [0.199–1.203]		0.119
**Vector species**					
*An. dirus* s.s.	>*An. minimus* s.s.		4.950 [1.423–17.223]		0.012
*An. dirus* s.s.	*An. barbirostris* s.l.		0.737 [0.243–2.230]		0.589
*An. dirus* s.s.	*An. sawadwongporni*		1.693 [0.545–5.264]		0.363
*An. dirus* s.s.	> *Ae. aegypti*		16.648 [3.597–77.049]		<0.001
*An. dirus* s.s.	*Ae. albopictus*		1.546 [0.516–4.632]		0.436
*An. dirus* s.s.	*An. maculatus* s.s.		ND^b^		ND
*An. minimus* s.s.	< *An. barbirostris* s.l.		0.149 [0.051–0.430]		<0.001
*An. minimus* s.s.	*An. sawadwongporni*		0.342 [0.110–1.064]		0.064
*An. minimus* s.s.	*Ae. aegypti*		3.363 [0.755–14.982]		0.112
*An. minimus* s.s.	< *Ae. albopictus*		0.312 [0.106–0.924]		0.036
*An. minimus* s.s.	*An. maculatus* s.s.		ND		ND
*An. barbirostris* s.l.	*An. sawadwongporni*		2.298 [0.860–6.145]		0.097
*An. barbirostris* s.l.	> *Ae. aegypti*		22.598 [5.639–90.556]		<0.001
*An. barbirostris* s.l.	*Ae. albopictus*		2.098 [0.824–5.345]		0.120
*An. barbirostris* s.l.	*An. maculatus* s.s.		ND		ND
*An. sawadwongporni*	> *Ae. aegypti*		9.833 [2.316–41.746]		0.002
*An. sawadwongporni*	*Ae. albopictus*		0.913 [0.346–2.408]		0.854
*An. sawadwongporni*	*An. maculatus* s.s.		ND		ND
*Ae. aegypti*	<*Ae. albopictus*		0.093 [0.023–0.380]		<001
*Ae. aegypti*	*An. maculatus* s.s.		ND		ND
*Ae. albopictus*	*An. maculatus* s.s.		ND		ND

Incidence Rate Ratios (IRR) with 95% confidence interval and p-values are reported.

a The Incidence Rate Ratio (IRR) indicates for how much more (if >1) or less (if <1) mosquitoes were collected in Group 1 as compared to Group 2. In the group with the highest number of mosquitoes collected, the protective efficacy of the tested repellents is the lowest.

b ND: Not Done. As *An. maculatus* s.s. was only collected on the negative control persons, and the model did not converge due to this event, this species was deleted from the analysis.

### Parity status in mosquitoes sensitive and insensitive to repellents

A total of 1040 mosquitoes were processed to define their parity status. The majority of dissected mosquitoes collected on the repellent treated persons were parous (66 parous out of the 71 (93%) dissected mosquitoes collected on repellent treated persons, versus 757 parous out of the 969 (78%) dissected mosquitoes collected on the control persons; p = 0.014 for pooled mosquito collections on all repellents; Table 5). Although parity rate differed significantly between the vector species (data not shown), no interaction was observed between species and treatment (p = 0.982).

**Table 5:** Logistic regression analysis of the effect of repellent treatment (including Ethanol as negative control) on the parity rate of the vector species.

Group 1 (parity rate)	Group 2 (parity rate)	OR*[95%CI]	p-value
picaridin 10% (93%)	> ethanol (78%)	3.177 [1.100–13.464]	0.061
picaridin 20% (89%)	ethanol (78%)	2.232 [0.602–14.468]	0.297
DEET 20% (100%)	ethanol (78%)	NA**	0.976
all repellents (93%)	> ethanol (78%)	3.271 [1.394–9.592]	0.014
Interaction species*treatment			0.982

Odds ratio's (OR) with 95% confidence intervals and p-values are reported.

* The Odds Ratio (OR) gives the odds of collecting a parous mosquito in Group 1 as compared to the odds of collecting a parous mosquito in Group 2 on persons treated with repellents. If OR<1 less mosquitoes were parous in Group1, if OR>1 more mosquitoes were parous in Group 1.

**No nulliparous mosquitoes were collected on DEET 20% treated persons, as such influencing the analysis to such an extent that NAs were generated in confidence limits of ORs.

### Infection rate in sensitive and insensitive mosquitoes

All of the anopheline mosquitoes were tested for the presence of *Plasmodium falciparum* (PF) or *P. vivax* (PV) sporozoites by sporozoite ELISA. None of the ELISA positive mosquitoes (10 *An. hyrcanus* for PV210, 2 *An. hyrcanus* for PF, 1 *An. maculatus s.s.* for PV210) were confirmed by PCR.

## Discussion

The present study is to our knowledge the most extensive study in Southeast Asia that measures the performance of picaridin repellents on wild anopheline and aedine vectors of malaria and arboviruses. The study was designed to measure the performance of the repellents over a five hour window only, as it was part of a project that measures the epidemiological impact of repellent use on malaria and arboviruses, *additional* to the use of ITNs. As such, it is important that the current gap in protection [4] due to early and outdoor biting vectors is filled.

In general, the repellents tested in this study performed very well, preventing more than 90% of mosquito bites on treated limbs, and with a median Complete Protection Time exceeding the five hours tested in this study. Beside coverage and regular compliance with treatment, repellent performance is an essential parameter for achieving an epidemiological impact on vector borne diseases. Based on a model [28], in low transmission or pre-elimination areas where most malaria transmission is residual, repellents with 90% entomological efficacy should reduce outdoor malaria transmission by up to 90% when used at a 100% compliance. Even if only about 50% of people comply with the regular treatment of an effective repellent, an additional reduction in transmission of 45% could be obtained. However this model does not consider a possible diversion of mosquitoes to non-repellent compliers [35].

In the present study, two repellent formulations were tested containing different concentrations of picaridin. The spray formulation, which has been shown to be the preferred repellent formulation by adults [36], contained 20% picaridin, which is considered a safe concentration for long-term use by adults [37]. The 10% picaridin lotion is better suited for application on children as the risk of spraying on sensitive areas of the body (e.g. eyes, nose, mouth, skin abrasions) is reduced [38], and the concentration is adapted to long-term use on children [37]. No significant difference in protective efficacy was observed between an ethanol solution of 20% DEET and the formulated 20% picaridin spray. The formulated 10% picaridin lotion was significantly less effective, although still more than 90% of mosquito bites were avoided. This confirms the findings of equal efficacy of ethanolic solutions of picaridin and DEET against anophelines and aedines obtained in laboratory tests [15], even if in the current study a commercially available picaridin formulation was compared to the ethanolic DEET solution. Also other field studies find similar protection rates for DEET and picaridin against several mosquito species in Malaysia [16], [17], Senegal [18], Australia [19], and the USA [20]. In contrast, a field study in Burkina Faso has shown that picaridin has a higher protection rate against several anophelines as compared to DEET [21]. The difference in findings between the current study and the study in Burkina Faso might be due to several factors. First, Cambodia has a different range of anopheline species as compared to Africa [39], [40], which could affect the results of this study, as differences in repellent sensitivity were observed between species (see further). Second, in the current study mosquito collections were only conducted during five hours after the application of the repellent. In the above mentioned study on African *Anopheles* vectors, picaridin always obtained the highest protection as compared to DEET at the end of the 10 hour exposure period [21]. The authors [21] also observed that picaridin remained on the treated limbs longer than DEET, suggesting that the longer-lasting protective efficacy observed with picaridin was presumably not due to higher sensitivity of *An. gambiae* s.l. to this compound, but rather to a longer residual effect on the skin. It has been shown that moderate levels of physical activity (jogging, stationary cycling) can result in a more than 40% decline in complete protection time of some repellents [41]. As such, the longer residual effect, together with the higher acceptance of picaridin as compared to DEET [14], could make picaridin a more appropriate repellent in vector control programs.

Additionally, as no decrease in repellent efficiency over time was observed (S1 & S2 Figs.), repellent sensitivity could be compared between genera and vector species. Differences were observed in the repellent performance between mosquito genera and species. About twice as many *Anopheles* spp. and *Aedes* spp. were collected on repellent treated persons as compared to *Mansonia* spp. and *Culex* spp. (Table 3), resulting in a difference in performance (percent repellency) of about 2% (Table 2). Therefore, the present study confirms the findings of laboratory tests, in which picaridin and DEET exhibit higher protection against *Culex* spp. as compared to aedines and anophelines [42]. In the current study, differences were also observed between vector species, with *Ae. aegypti* and *An. minimus* s.s. being the most sensitive to the used repellents, and *An. barbirostris* s.l. the least, although these differences were less for DEET 20%. Moreover both repellents are more effective against *Ae. aegypti* than *Ae. albopictus*. Differences in repellent sensitivity were also observed between closely related species. Although sample size did not allow to detect differences between *An. maculatus* s.s. and *An. sawadwongporni*, it is striking that no *An. maculatus* s.s. were collected on repellent treated persons, whereas repellent insensitivity was observed in *An. sawadwongporni*. It has been suggested that for field studies the repellent performance cannot be compared between mosquito species [21], due to decreases in repellent efficiency over time, and concurrent differences in biting peaks or biting densities between species. As such, measuring the effective dose for each species in experimental conditions [43] would provide a more precise estimate for comparing the sensitivity between mosquito populations, but the number of mosquito species available in insectary colonies are limited. Moreover, each mosquito colony passes a bottleneck when established in the laboratory, resulting in degeneration of the gene pool and loss or changes within its behavioural repertoire [44], making colonized mosquitoes not representative of field populations. Therefore, field studies can provide additional information. As mentioned above, in the current field study no decrease in repellent efficiency was observed over the five hour experiment. The differences observed in performance between mosquito genera or species were therefore not likely to be due to differences in biting times or biting densities. This is illustrated by the fact that the repellents performed better against *Culex* spp., with the highest biting densities until five hours after repellent application, as compared to *Aedes* spp., with lower biting densities and an early biting peak. Also, *Ae. aegypti* and *Ae. albopictus* had similar biting dynamics (Fig. 1), but differed in their repellent sensitivity. Further, the greatest repellent insensitivity was observed in *An. barbirostris* with a biting activity which was almost constant between 2 and 5 hours after treatment, and which was only present at low densities. It has been suggested that feeding avidity can also influence repellent insensitivity [43], [45]. As such vectors with a more anthropophilic trend might exhibit higher repellent insensitivity. In the current study, this is indeed the case for the very anthropophilic vector *An. dirus*. However, *An. barbirostris*, which is usually considered a more zoophilic mosquito [46], showed the highest repellent insensitivity, suggesting other mechanisms, e.g. molecular variations in odour receptors targeted by the repellents. Repellent insensitivity in certain species has indeed been observed in previous studies [17], [21], [43], and can be selected in the laboratory as shown experimentally for *Ae. aegypti* [47], and for *An. dirus* of which a colony established from Chonburi (Thailand) in 1968 was tolerant to DEET-concentrations lower than 30% [48]. Unfortunately, the exact mode of action and molecular targets of DEET and picaridin are not yet completely understood, so molecular explanations for the observed genus- and species-specific differences in repellent sensitivity cannot be provided. DEET and picaridin are believed to have an effect on the olfactory system consisting of odorant receptors (ORs) that need a common co-receptor (ORCO), and of ionotropic receptors (IR) [49]. Recent data support the hypothesis that DEET alters the fine-tuning of the insect olfactory system [50], as well as triggers a direct response of ORs [51], [52], ORCO [52]–[54] or IRs [55]. ORCO and IR40a orthologues are conserved across many insect species, possibly explaining the wide action of DEET as repellent for many insect species [55], [56]. Few research has been carried out on the mode of action of picaridin, but it has been suggested that picaridin might also target the co-receptor ORCO [57]. Further research to detect genetic alterations (e.g. mutation, duplication, upregulation) in these receptors between the currently collected sensitive and insensitive mosquitoes as such could provide key knowledge on the mode of action of both repellents.

It has been suggested that the infection status of a mosquito can alter its blood feeding behaviour [58], and that pathogen infected mosquitoes might respond differently to repellents [59]. In this study however, no malaria sporozoites were detected in any of the collected mosquitoes, which is not surprising regarding the low malaria endemicity (<5%). In previous field studies no significant differences were found in the proportion of anophelines harbouring *Plasmodium* sporozoites landing on control or repellent treated individuals [21], [60]. Experimental infections with the four serotypes of Dengue Virus did not alter the responses to DEET of *Ae. aegypti* and *Ae. albopictus* [61], although experimental disseminated Sindbis Virus infection in *Ae. aegypti* did significantly reduce its time to first bite on DEET and picaridin treated artificial blood meal substrates [59]. As such, until the latter finding is confirmed in the field, it can be assumed that a repellent reducing the number of vector bites, will also reduce the number of infectious bites. Surprisingly, in the present study a higher proportion of parous mosquitoes landed on repellent treated legs as compared to control persons, and this for all vector species involved. This might be related to differences in host avidity between parous and nulliparous mosquitoes as experimentally shown for *Ae. albopictus* [45]. Despite the statistical analysis being based on a low number of repellent insensitive mosquitoes, it is worth mentioning as the vectorial capacity for a population of vectors is highly dependent on its age structure [62]. As such, older (parous) mosquitoes are more likely to harbour infectious pathogens given the extrinsic incubation period of the pathogens in the vector. Therefore, parity status of vector populations should be systematically documented in future field evaluations of repellents and other vector control tools.

In conclusion, field evaluation of formulated picaridin repellents shows that the 20% spray formulation performs equally well as the 20% DEET solution, both protecting users from more than 98% of the mosquito bites in the study area. Over the five hour test period, no significant decline in the repellents' efficacy was observed, showing that these repellents can be used as additional personal protection tools against early and outdoor biting vectors. The heterogeneity in repellent sensitivity between mosquito genera and vector species could however impact the efficacy of repellents in public health programs. Considering its excellent performance and potential to protect against early and outdoor biting vectors, as well as its higher acceptability as compared to DEET, picaridin is an appropriate product to evaluate the epidemiological impact of large scale use of topical repellents on arthropod borne diseases.

## Supporting Information

S1 Fig Repellent performance per collection hour (1st, 2nd, 3rd, 4th, 5th) expressed as the percent (%) repellency (the relative proportion of mosquitoes repelled by the used repellent) for *Aedes* spp. (A), *Anopheles* spp. (B), *Culex* spp. (C) and *Mansonia* spp. (D), shown per repellent (picaridin 10%, picaridin 20% and DEET 20%).
(TIF)

S2 Fig Repellent performance per collection hour (1st, 2nd, 3rd, 4th, 5th) expressed as the percent (%) Repellency (the relative proportion of mosquitoes repelled by the used repellent) for *Ae. aegypti* (A), *Ae. albopictus* (B), *An. barbirostris s.l.* (C), *An. dirus s.s.* (D), *An. maculatus* s.s. (E), *An. minimus* s.s. (F), and *An. sawadwongporni* (G), shown per repellent (picaridin 10%, picaridin 20% and DEET 20%).
(TIF)

S3 Fig Repellent performance expressed as the percent (%) repellency (the relative proportion of mosquitoes repelled by the used repellent) for picaridin 10%, picaridin 20% and DEET 20%, per mosquito genus (A) or selected vector species (B).
(TIF)

## References

[1] McGraw EA, O'Neill SL (2013) Beyond insecticides: new thinking on an ancient problem. Nat Rev Microbiol 11: 181–193. 23411863 10.1038/nrmicro2968

[2] Tolle MA (2009) Mosquito-borne diseases. Curr Probl Pediatr Adolesc Health Care 39: 97–140. 19327647 10.1016/j.cppeds.2009.01.001

[3] Gubler DJ (2002) The global emergence/resurgence of arboviral diseases as public health problems. Arch Med Res 33: 330–342. 12234522 10.1016/s0188-4409(02)00378-8

[4] Durnez L, Coosemans M (2013) Residual Transmission of Malaria: An Old Issue for New Approaches. In: Manguin S, editor. *Anopheles* mosquitoes - New insights into malaria vectors.Intech.pp. 671–704. Available: http://www.intechopen.com/books/anopheles-mosquitoes-new-insights-into-malaria-vectors/residual-transmission-of-malaria-an-old-issue-for-new-approaches.

[5] WHO (2013) World Malaria Report 2013. Geneva. Available: http://www.who.int/malaria/publications/world_malaria_report_2013/en/.

[6] Russell TL, Govella NJ, Azizi S, Drakeley CJ, Kachur SP, et al (2011) Increased proportions of outdoor feeding among residual malaria vector populations following increased use of insecticide-treated nets in rural Tanzania. Malar J 10: 80. 21477321 10.1186/1475-2875-10-80 PMC3084176

[7] Van Bortel W, Trung HD, Hoi LX, Van Ham N, Van Chut N, et al (2010) Malaria transmission and vector behaviour in a forested malaria focus in central Vietnam and the implications for vector control. Malar J 9: 373. 21182774 10.1186/1475-2875-9-373 PMC3224380

[8] Durnez L, Mao S, Denis L, Roelants P, Sochantha T, et al (2013) Outdoor malaria transmission in forested villages of Cambodia. Malar J 12: 329. 24044424 10.1186/1475-2875-12-329 PMC3848552

[9] Delacollette C, D'Souza C, Christophel E, Thimasarn K, Abdur R, et al (2009) Malaria trends and challenges in the Greater Mekong Subregion. Southeast Asian J Trop Med Public Health 40: 674–691. 19842400

[10] Thisyakorn U, Thisyakorn C (2014) Latest developments and future directions in dengue vaccines. Ther Adv vaccines 2: 3–9. 24757522 10.1177/2051013613507862 PMC3991153

[11] Caglioti C, Lalle E, Castilletti C, Carletti F, Capobianchi MR, et al (2013) Chikungunya virus infection: an overview. New Microbiol 36: 211–227. 23912863

[12] Bettadapura J, Herrero LJ, Taylor A, Mahalingam S (2013) Approaches to the treatment of disease induced by chikungunya virus. Indian J Med Res 138: 762–765. 24434329 PMC3928707

[13] Thavara U, Tawatsin A, Chansang C, Kong-ngamsuk W, Paosriwong S, et al (2001) Larval occurrence, oviposition behavior and biting activity of potential mosquito vectors of dengue on Samui Island, Thailand. J Vector Ecol 26: 172–180. 11813654

[14] Moore SJ, Debboun M (2007) History of Insect Repellents. In: Debboun M, Frances SP, Strickman D, editors. Insect Repellents.Principles, Methods, and Uses. New York: CRC Press. pp. 3–29.

[15] Badolo A, Ilboudo-Sanogo E, Ouédraogo AP, Costantini C (2004) Evaluation of the sensitivity of *Aedes aegypti* and *Anopheles gambiae* complex mosquitoes to two insect repellents: DEET and KBR 3023. Trop Med Int Health 9: 330–334. 14996361 10.1111/j.1365-3156.2004.01206.x

[16] Yap HH, Jahangir K, Chong AS, Adanan CR, Chong NL, et al (1998) Field efficacy of a new repellent, KBR 3023, against *Aedes albopictus* (Skuse) and *Culex quinquefasciatus* (Say) in a tropical environment. J Vector Ecol 23: 62–68. 9673931

[17] Yap HH, Jahangir K, Zairi J (2000) Field efficacy of four insect repellent products against vector mosquitoes in a tropical environment. J Am Mosq Control Assoc 16: 241–244. 11081653

[18] Uzzan B, Konate L, Diop A, Nicolas P, Dia I, et al (2009) Efficacy of four insect repellents against mosquito bites: a double-blind randomized placebo-controlled field study in Senegal. Fundam Clin Pharmacol 23: 589–594. 19744033 10.1111/j.1472-8206.2009.00731.x

[19] Frances SP, Waterson DGE, Beebe NW, Cooper RD (2004) Field evaluation of repellent formulations containing deet and picaridin against mosquitoes in Northern Territory, Australia. J Med Entomol 41: 414–417. 15185943 10.1603/0022-2585-41.3.414

[20] Barnard DR, Bernier UR, Posey KH, Xue R-D (2002) Repellency of IR3535, KBR3023, para-menthane-3,8-diol, and deet to black salt marsh mosquitoes (Diptera: Culicidae) in the Everglades National Park. J Med Entomol 39: 895–899. 12495189 10.1603/0022-2585-39.6.895

[21] Costantini C, Badolo A, Ilboudo-Sanogo E (2004) Field evaluation of the efficacy and persistence of insect repellents DEET, IR3535, and KBR 3023 against *Anopheles gambiae* complex and other Afrotropical vector mosquitoes. Trans R Soc Trop Med Hyg 98: 644–652. 15363644 10.1016/j.trstmh.2003.12.015

[22] Hill N, Lenglet A, Arnéz AM, Carneiro I (2007) Plant based insect repellent and insecticide treated bed nets to protect against malaria in areas of early evening biting vectors: double blind randomised placebo controlled clinical trial in the Bolivian Amazon. BMJ 335: 1023. 17940319 10.1136/bmj.39356.574641.55 PMC2078668

[23] Dutta P, Khan AM, Khan SA, Borah J, Sharma CK, et al (2011) Malaria control in a forest fringe area of Assam, India: a pilot study. Trans R Soc Trop Med Hyg 105: 327–332. 21549402 10.1016/j.trstmh.2011.02.008

[24] Rowland M, Downey G, Rab A, Freeman T, Mohammad N, et al (2004) DEET mosquito repellent provides personal protection against malaria: a household randomized trial in an Afghan refugee camp in Pakistan. Trop Med Int Health 9: 335–342. 14996362 10.1111/j.1365-3156.2004.01198.x

[25] Dadzie S, Boakye D, Asoala V, Koram K, Kiszewski A, et al (2013) A community-wide study of malaria reduction: evaluating efficacy and user-acceptance of a low-cost repellent in northern Ghana. Am J Trop Med Hyg 88: 309–314. 23249683 10.4269/ajtmh.2012.12-0370 PMC3583322

[26] Prakash A, Bhattacharyya DR, Mohapatra PK, Barua U, Phukan A, et al (2003) Malaria control in a forest camp in an oil exploration area of Upper Assam. Natl Med J India 16: 135–138. 12929855

[27] Chen-Hussey V, Carneiro I, Keomanila H, Gray R, Bannavong S, et al (2013) Can topical insect repellents reduce malaria? A cluster-randomised controlled trial of the insect repellent N, N-diethyl-m-toluamide (DEET) in Lao PDR. PLoS One 8: e70664. 23967083 10.1371/journal.pone.0070664 PMC3743820

[28] Kiszewski AE, Darling ST (2010) Estimating a mosquito repellent ' s potential to reduce malaria in communities. J Vector Borne Dis 47: 217–221. 21178214

[29] WHO (2009) Guidelines for efficacy testing of mosquito repellents for human skin. Geneva. Available: http://whqlibdoc.who.int/hq/2009/WHO_HTM_NTD_WHOPES_2009.4_eng.pdf.

[30] Moore SJ, Davies CR, Hill N, Cameron MM (2007) Are mosquitoes diverted from repellent-using individuals to non-users? Results of a field study in Bolivia. Trop Med Int Health 12: 532–539. 17445144 10.1111/j.1365-3156.2006.01811.x

[31] MR4 (2011) Methods in Anopheles Research. Malaria Research and Reference Reagent Resource Center. Available: http://www.mr4.org/Publications/MethodsinAnophelesResearch.aspx.

[32] Durnez L, Van Bortel W, Denis L, Roelants P, Veracx A, et al (2011) False positive circumsporozoite protein ELISA: a challenge for the estimation of the entomological inoculation rate of malaria and for vector incrimination. Malar J 10: 195. 21767376 10.1186/1475-2875-10-195 PMC3160429

[33] Newcombe RG (1998) Two-sided confidence intervals for the single proportion: comparison of seven methods. Stat Med 17: 857–872. 9595616 10.1002/(sici)1097-0258(19980430)17:8<857::aid-sim777>3.0.co;2-e

[34] O'Hara RB, Kotze DJ (2010) Do not log-transform count data. Methods Ecol Evol 1: 118–122.

[35] Maia MF, Onyango SP, Thele M, Simfukwe ET, Turner EL, et al (2013) Do topical repellents divert mosquitoes within a community? Health equity implications of topical repellents as a mosquito bite prevention tool. PLoS One 8: e84875. 24376852 10.1371/journal.pone.0084875 PMC3869929

[36] Frances SP, Debboun M (2007) User acceptability: public perceptions of insect repellents. In: Debboun M, Frances SP, Strickman D, editors. Insect Repellents.Principles, Methods, and Uses. Boca Raton: CRC Press. p. 495.

[37] Boulanger N, de Gentile L (2012) Les répulsifs cutanés. In: Duvallet G, de Gentile L, editors. Protection personnelle antivectorielle. Marseille: IRD éditions. p. 352.

[38] Xue R-D, Ali A, Day JF (2007) Commercially available insect repellents and criteria for their use. In: Debboun M, Frances SP, Strickman D, editors. Insect Repellents.Principles, Methods, and Uses.Boca Raton: CRC Press. p. 495.

[39] Sinka ME, Bangs MJ, Manguin S, Coetzee M, Mbogo CM, et al (2010) The dominant *Anopheles* vectors of human malaria in Africa, Europe and the Middle East: occurrence data, distribution maps and bionomic précis. Parasit Vectors 3: 117. 21129198 10.1186/1756-3305-3-117 PMC3016360

[40] Sinka ME, Bangs MJ, Manguin S, Chareonviriyaphap T, Patil AP, et al (2011) The dominant *Anopheles* vectors of human malaria in the Asia-Pacific region: occurrence data, distribution maps and bionomic précis. Parasit Vectors 4: 89. 21612587 10.1186/1756-3305-4-89 PMC3127851

[41] Schofield S, Tepper M, Gadawski R (2007) Laboratory and field evaluation of the impact of exercise on the performance of regular and polymer-based deet repellents. J Med Entomol 44: 1026–1031. 18047202 10.1603/0022-2585(2007)44[1026:lafeot]2.0.co;2

[42] Lupi E, Hatz C, Schlagenhauf P (2013) The efficacy of repellents against *Aedes*, *Anopheles*, *Culex* and *Ixodes* spp. - a literature review. Travel Med Infect Dis 11: 374–411. 24201040 10.1016/j.tmaid.2013.10.005

[43] Curtis CF, Lines JD, Ijumba J, Callaghan A, Hill N, et al (1987) The relative efficacy of repellents against mosquito vectors of disease. Med Vet Entomol 1: 109–119. 2908762 10.1111/j.1365-2915.1987.tb00331.x

[44] Spitzen J, Takken W (2005) Malaria mosquito rearing - maintaining quality and quantity of laboratory-reared insects. Proc Neth Entomol Soc Meet. p 16: 95–100.

[45] Xue RD, Barnard DR (1996) Human host avidity in *Aedes albopictus*: influence of mosquito body size, age, parity, and time of day. J Am Mosq Control Assoc 12: 58–63. 8723259

[46] Trung HD, Bortel W Van, Sochantha T, Keokenchanh K, Briët OJT, et al (2005) Behavioural heterogeneity of *Anopheles* species in ecologically different localities in Southeast Asia: a challenge for vector control. Trop Med Int Health 10: 251–262. 15730510 10.1111/j.1365-3156.2004.01378.x

[47] Stanczyk NM, Brookfield JFY, Ignell R, Logan JG, Field LM (2010) Behavioral insensitivity to DEET in *Aedes aegypti* is a genetically determined trait residing in changes in sensillum function. Proc Natl Acad Sci USA 107: 8575–8580. 20439757 10.1073/pnas.1001313107 PMC2889326

[48] Frances SP, Eikarat N, Sripongsai B, Eamsila C (1993) Response of *Anopheles dirus* and *Aedes albopictus* to repellents in the laboratory. J Am Mosq Control Assoc 9: 474–476. 8126487

[49] Hallem EA, Dahanukar A, Carlson JR (2006) Insect odor and taste receptors. Annu Rev Entomol 51: 113–135. 16332206 10.1146/annurev.ento.51.051705.113646

[50] Pellegrino M, Steinbach N, Stensmyr MC, Hansson BS, Vosshall LB (2011) A natural polymorphism alters odour and DEET sensitivity in an insect odorant receptor. Nature 478: 511–514. 21937991 10.1038/nature10438 PMC3203342

[51] Fradin MS (1998) Mosquitoes and Mosquito Repellents: A Clinician's Guide. 128: 931–940. 10.7326/0003-4819-128-11-199806010-00013 9634433

[52] Liu C, Pitts RJ, Bohbot JD, Jones PL, Wang G, et al (2010) Distinct olfactory signaling mechanisms in the malaria vector mosquito *Anopheles gambiae* . PLoS Biol 8: 1–17. 10.1371/journal.pbio.1000467 PMC2930861 20824161

[53] Ditzen M, Pellegrino M, Vosshall LB (2008) Insect odorant receptors are molecular targets of the insect repellent DEET. Science 319: 1838–1842. 18339904 10.1126/science.1153121

[54] DeGennaro M, McBride CS, Seeholzer L, Nakagawa T, Dennis EJ, et al (2013) Orco mutant mosquitoes lose strong preference for humans and are not repelled by volatile DEET. Nature 498: 487–491. 23719379 10.1038/nature12206 PMC3696029

[55] Kain P, Boyle SM, Tharadra SK, Guda T, Pham C, et al (2013) Odour receptors and neurons for DEET and new insect repellents. Nature 502: 507–514. 24089210 10.1038/nature12594 PMC3927149

[56] Jones WD, Nguyen T-AT, Kloss B, Lee KJ, Vosshall LB (2005) Functional conservation of an insect odorant receptor gene across 250 million years of evolution. Curr Biol 15: R119–21. 10.1016/j.cub.2005.02.007 15723778

[57] Bohbot JD, Dickens JC (2010) Insect repellents: modulators of mosquito odorant receptor activity. PLoS One 5: e12138. 20725637 10.1371/journal.pone.0012138 PMC2920324

[58] Cornet S, Nicot A, Rivero A, Gandon S (2013) Malaria infection increases bird attractiveness to uninfected mosquitoes. Ecol Lett 16: 323–329. 23205903 10.1111/ele.12041

[59] Qualls WA, Day JF, Xue R, Bowers DF (2012) Altered behavioral responses of Sindbis virus-infected *Aedes aegypti* (Diptera: Culicidae) to DEET and non-DEET based insect repellents. Acta Trop 122: 284–290. 22289669 10.1016/j.actatropica.2012.01.012

[60] Copeland RS, Walker TW, Robert LL, Githure JI, Wirtz RA, et al (1995) Response of wild *Anopheles funestus* to repellent-protected volunteers is unaffected by malaria infection of the vector. J Am Mosq Control Assoc 11: 438–440. 8825504

[61] Frances SP, Sithiprasasna R, Linthicum KJ (2011) Laboratory evaluation of the response of *Aedes aegypti* and *Aedes albopictus* uninfected and infected with dengue virus to deet. J Med Entomol 48: 334–336. 21485370 10.1603/me10120

[62] Mouchet J, Carnevale P, Manguin S (2008) Biodiversity of Malaria in the World. John Libbey Eurotext.

