# Supplementary figures and images for: Field Evaluation of Picaridin Repellents Reveals Differences in Repellent Sensitivity between Southeast Asian Vectors of Malaria and Arboviruses
Source: PLoS Negl Trop Dis. 2014 Dec 18;8(12):e3326. doi: 10.1371/journal.pntd.0003326 (PMC4270489; doi:10.1371/journal.pntd.0003326)

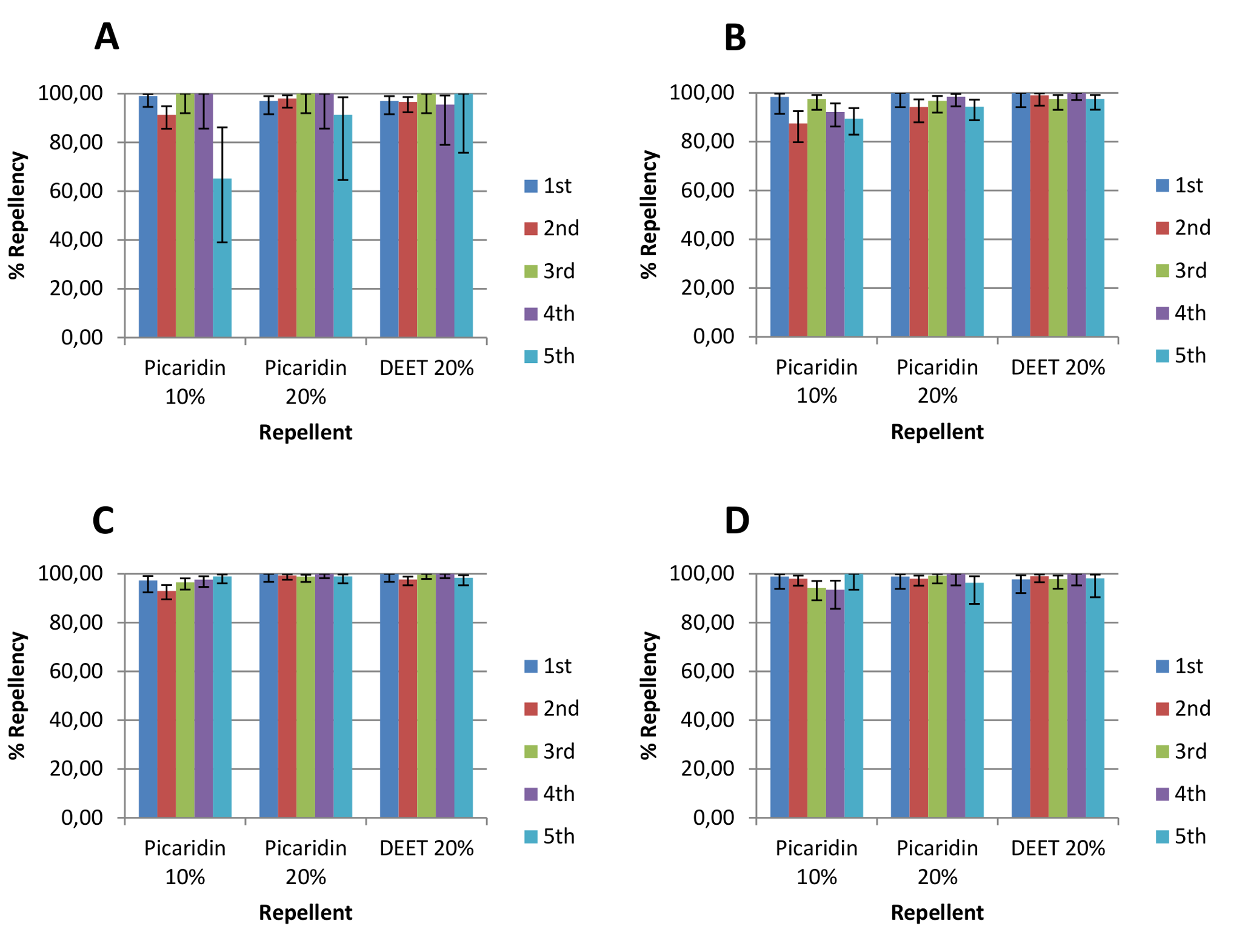

Supplement: S1 Fig — Repellent performance per collection hour (1st, 2nd, 3rd, 4th, 5th) expressed as the percent (%) repellency (the relative proportion of mosquitoes repelled by the used repellent) for Aedes spp. (A), Anopheles spp. (B), Culex spp. (C) and Mansonia spp. (D), shown per repellent (picaridin 10%, picaridin 20% and DEET 20%). (TIF) [file pntd.0003326.s001.tif]

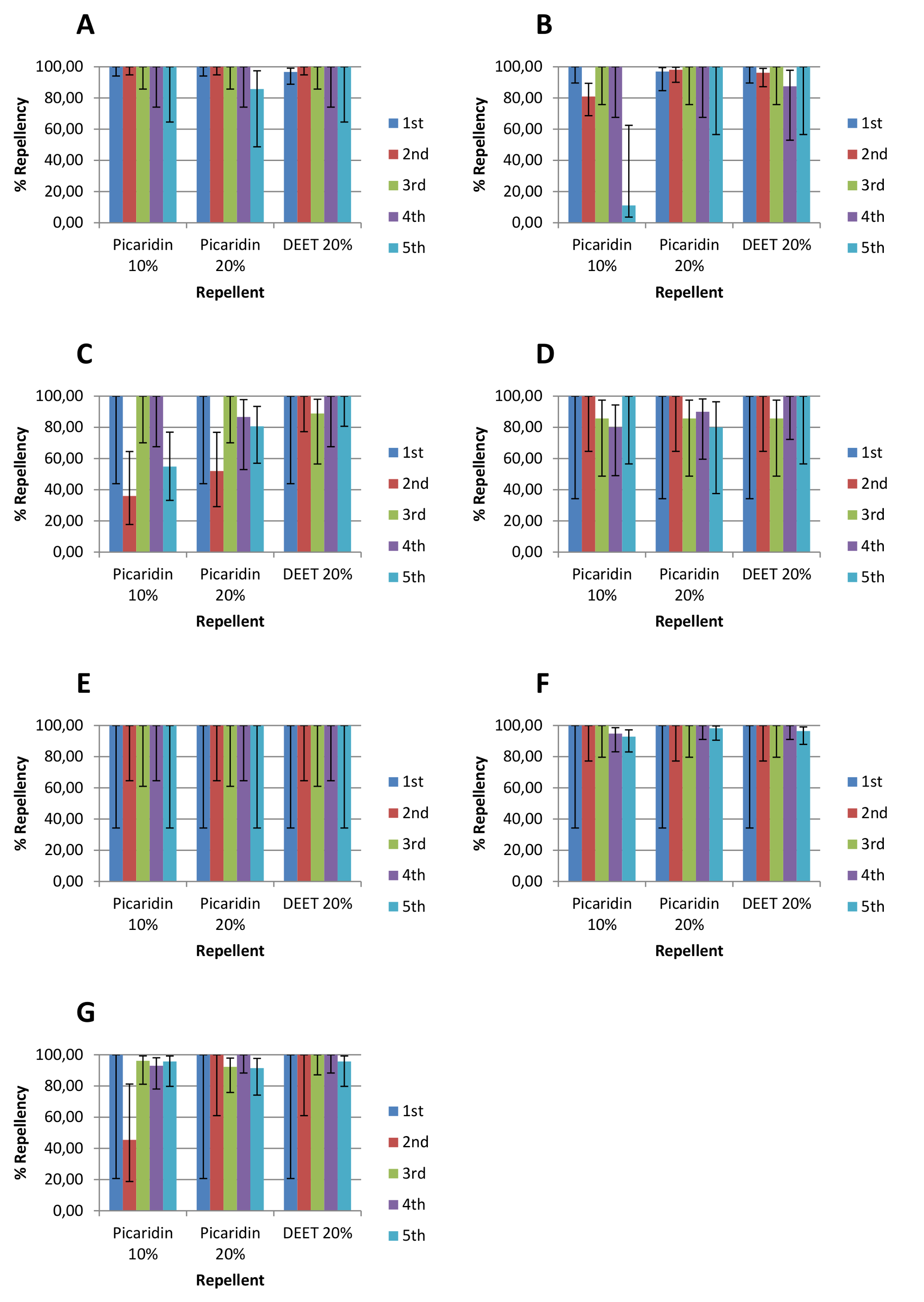

Supplement: S2 Fig — Repellent performance per collection hour (1st, 2nd, 3rd, 4th, 5th) expressed as the percent (%) Repellency (the relative proportion of mosquitoes repelled by the used repellent) for Ae. aegypti (A), Ae. albopictus (B), An. barbirostris s.l. (C), An. dirus s.s. (D), An. maculatus s.s. (E), An. minimus s.s. (F), and An. sawadwongporni (G), shown per repellent (picaridin 10%, picaridin 20% and DEET 20%). (TIF) [file pntd.0003326.s002.tif]

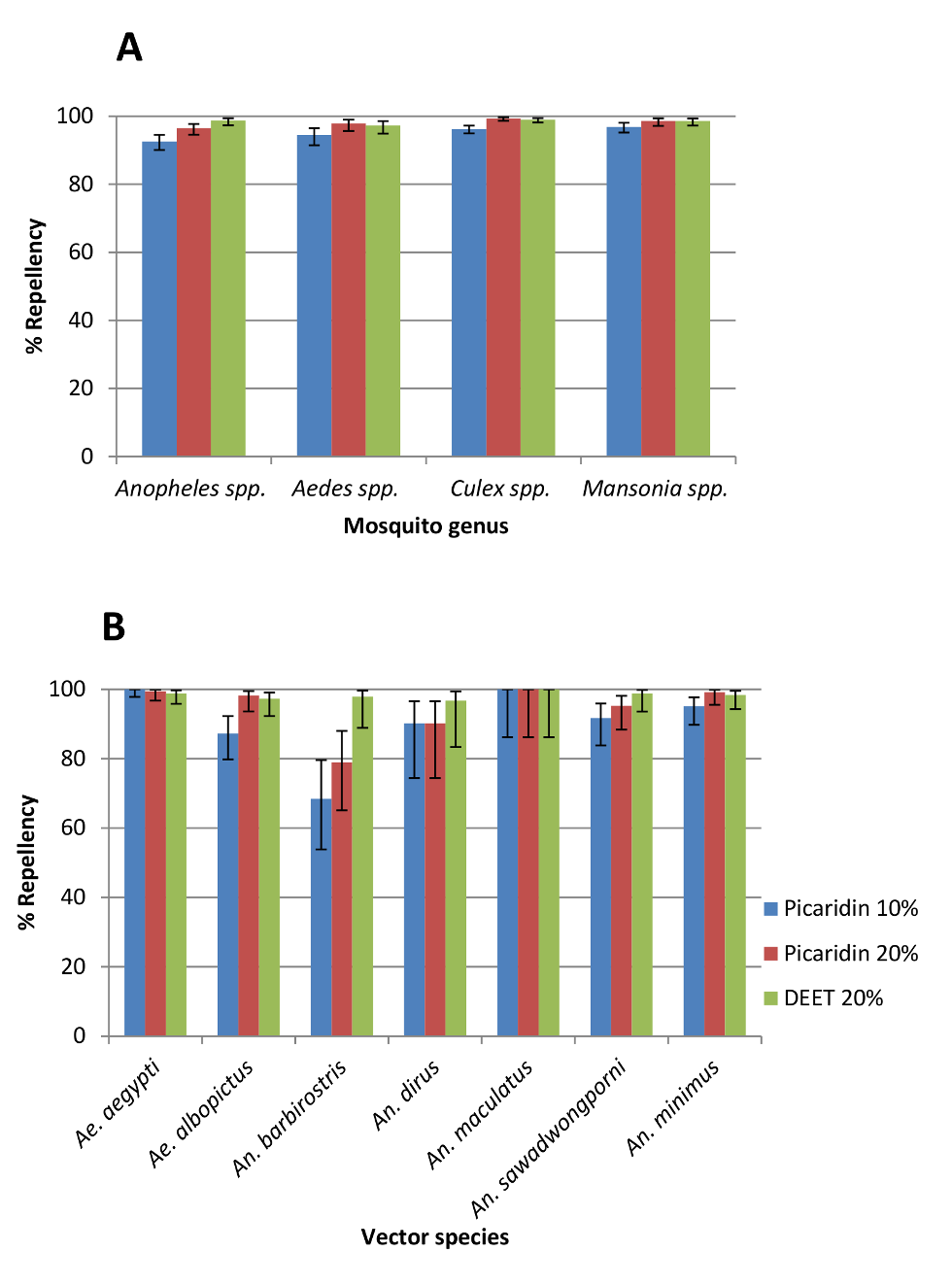

Supplement: S3 Fig — Repellent performance expressed as the percent (%) repellency (the relative proportion of mosquitoes repelled by the used repellent) for picaridin 10%, picaridin 20% and DEET 20%, per mosquito genus (A) or selected vector species (B). (TIF) [file pntd.0003326.s003.tif]
